# Home-based biofeedback with local anal electrical stimulation for fecal incontinence in women without sphincter structural defects

**DOI:** 10.3389/fmed.2026.1835226

**Published:** 2026-06-12

**Authors:** Peter Liptak, Adam Lukac, Jan Mikler, Peter Banovcin

**Affiliations:** 1 Clinic of Internal Medicine-Gastroenterology, University Hospital Martin, Martin, Slovakia; 2 Jessenius Faculty of Medicine in Martin, Comenius University in Bratislava, Bratislava, Slovakia; 3 Clinic of Children and Adolescents, University Hospital Martin, Martin, Slovakia

**Keywords:** biofeedback, electrical stimulation, fecal incontinence, therapy, women

## Abstract

**Background:**

Fecal incontinence (FI) is a prevalent and distressing condition with restricted access to specialized biofeedback therapy. Home-based biofeedback devices constitute a potential solution to enhance treatment accessibility; however, evidence from sham-controlled trials assessing their physiological effects remains scarce.

**Methods:**

We conducted a single-center exploratory pilot study with a sham control involving women diagnosed with fecal incontinence. Baseline and post-treatment assessments included anorectal manometry, endoanal ultrasound, and evaluations of clinical symptoms. Subjects were allocated to either the intervention or sham therapy group and performed home training sessions six times per week over a period of 12 weeks. Adherence to the training regimen was monitored via a mobile application.

**Results:**

A total of 67 patients were screened, and 14 were assigned to intervention (*n* = 8) or sham control (*n* = 6). Twelve participants completed the study (six in each group). Improvements in anorectal manometric parameters were observed in the intervention group compared with the sham group, although the differences did not reach statistical significance. Clinical symptom scores also showed greater improvement in the intervention group. App-based monitoring demonstrated high adherence to home training, with an average of 5.5 training sessions per week and an overall adherence rate of 91%.

**Conclusion:**

Home-based electrical biofeedback therapy was associated with positive trends in both anorectal physiological parameters and clinical symptom scores in women with fecal incontinence. These preliminary findings provide a basis for further investigation into home-based rehabilitation support strategies within controlled pelvic floor rehabilitation programs through larger randomized controlled trials.

## Introduction

Fecal incontinence (FI) is a fairly common condition characterized by the involuntary passage of fecal matter (1). Treating FI poses many challenges and often yields unsatisfactory outcomes for patients. Consequently, management should be tailored to each individual’s symptoms (2).

Biofeedback training is recommended when a motor function disorder is diagnosed and patients can learn voluntary control of their responses, potentially leading to further improvement.

The American Neurogastroenterology and Motility Society (ANMS) and the European Society of Neurgastroenterology and Motility (ESNM) recommend biofeedback for the short-term and long-term treatment of fecal incontinence in adults (3). Most biofeedback training occurs in outpatient settings at specialized centers, meaning this treatment is not widely accessible.

Due to the time-consuming and costly nature of ambulatory biofeedback, there is a growing trend toward developing home biofeedback devices and protocols. However, only a limited number of studies on this innovative approach have been published, and their scientific rigor varies (4).

Therefore, we decided to perform a pilot exploratory feasibility study with sham control on a well-defined cohort of patients to evaluate the mechanistic and clinical effects of biofeedback on symptoms of fecal incontinence and the anal sphincter function.

## Methods

A single-center pilot, prospective feasibility and exploratory study with sham control was conducted in a tertiary gastroenterology center from October 2024 to March 2026. The study was approved by the local Ethics Committee (registered with the US Office for Human Research Protection as IRB00005636) with approval number 76/2024, and was conducted in accordance with the CONSORT 2010 standards for pilot and feasibility trials (5) (checklist in the Supplementary material). All participants provided written informed consent. Inclusion criteria were age>18, female sex, distinctive phenotype of fecal incontinence (urge or passive) with duration of at least 6 months, and no medical history of surgery or injury in the anorectal region. The exclusion criteria were age <18 years, inability to follow study protocol, incontinence combined with constipation or a non-distinctive type of incontinence, surgical procedure in the anal region in the past, known clinically relevant neurological disorder, and clinically significant hemorrhoidal disease. The participants were allocated to either the intervention or sham group through simple randomization, utilizing a pre-generated allocation sequence developed via an online randomization tool^1^ and subsequently exported to Microsoft Excel (USA). The sequence comprised randomly ordered group assignments (sham, sham, intervention, etc.), and participants were enrolled sequentially according to this list. Given the preliminary nature of the study, neither block randomization nor stratification was employed, and allocation concealment was not formally instituted.

All participants were evaluated using 3D HD anorectal manometry during the screening process. The measured parameters included resting mean pressure (pressure during the rest phase, representing the basal pressure of the internal and external sphincters), rest maximum pressure (the highest pressure recorded during the resting phase), voluntary maximum pressure (the maximum pressure achieved voluntarily by the patient through contraction of the external anal sphincter), and the duration of voluntary maximum pressure. During the rest phase, patients were instructed to relax their sphincters for 4 min (3 min for accommodation and 1 min for measurement). Afterwards, they were actively instructed by the healthcare provider to squeeze the sphincter four times (three times for 5 s and once for 30 s), which was used to evaluate voluntary maximum pressure and its duration. Furthermore, patients were subsequently examined with endoanal ultrasonography to confirm the structural integrity of the external and internal sphincters. All patients completed the self-reporting St. Marks Incontinence Score questionnaire and the Fecal Incontinence Severity Index (FISI) (6) at both the primary and follow-up visits.

After successful enrollment, the participants were assigned in the interventional or sham device. Both the intervention and sham groups were equipped with the home biofeedback device (NeuroTrac Myo Plus, Verity Medical, Ireland), connected to an intra-anal probe inserted during the home training session to a depth of approximately 1.5 to 2 cm into the anal canal. This probe delivered electrical stimulation directly to the entire circumference of the anal sphincter complex via conductive metal plates on its surface. The probe was held in a fixed position during the training session. Both groups were instructed to train six times weekly, once daily, for 26 min. The training lasted 12 weeks. The intervention group received 6 min of continuous stimulation (frequency 3 Hz, pulse width 330 μs), followed by 20 min of work/rest cycles (frequency 35 Hz, pulse width 300 μs, work phase 5 s, rest phase 5 s, ramp-up 0.5 s). The sham group used an EMG program that displayed EMG feedback with beeping sounds mimicking stimulation. Participants were instructed not to perform any pelvic muscle exercises during the training sessions.

To ensure adherence, a simple free mobile application was provided, developed on jotform.com. Through this app, all participants who agreed to have it installed on their smartphones received weekly push notifications to report in the app: 1. how often they trained, 2. what stimulation current they used, and if they had any questions about the training, they could message the study team. All data were anonymously collected on the platform for further analysis. Each patient was assigned a study code, and only one member of the study team could match the actual patient to the code used in the application. This ensured complete anonymization of the patients’ data.

The follow-up visit was scheduled after 12 weeks of training, during which the questionnaires were completed again and the same 3D HD manometry protocol was performed. For patients in the sham group, the option to continue with the studied intervention was offered to ensure that every patient had access to the same therapy.

Primary endpoint was the change in resting maximum anal pressure, voluntary maximum pressure. Secondary endpoints were changes in the St. Mark’s score and fecal incontinence severity score (FISI).

The data storage and statistical analysis were performed using Excel (Microsoft, USA). Wilcoxon signed-rank test was used to compare paired pre- and post-therapy measurements within each group, and Mann–Whitney U test was used for between-group comparisons. Effect sizes for non-parametric tests were calculated using the correlation coefficient *r* and were interpreted as small (0.1), moderate (0.3), and large (0.5).

## Results

Altogether, 67 patients were screened for eligibility. Of these, 14 (20.8%) were assigned to either the intervention group (*n* = 8) or the sham group (*n* = 6). All participants in the sham group completed the 12-week training program, while six participants in the intervention group completed it. Two participants from the intervention group did not complete the study—one due to personal reasons and another because of travel out of the country. Consequently, 12 patients—six from each group—were included in the final evaluation. All participants were women (female-to-male ratio 12:0), with an average age of 63.7 years (61.3 in the intervention group and 66.2 in the sham group). The average body mass index was 26.1 in the intervention group and 25.7 in the sham group. No adverse events were reported.

### Manometric outcomes

**Figure 1:**
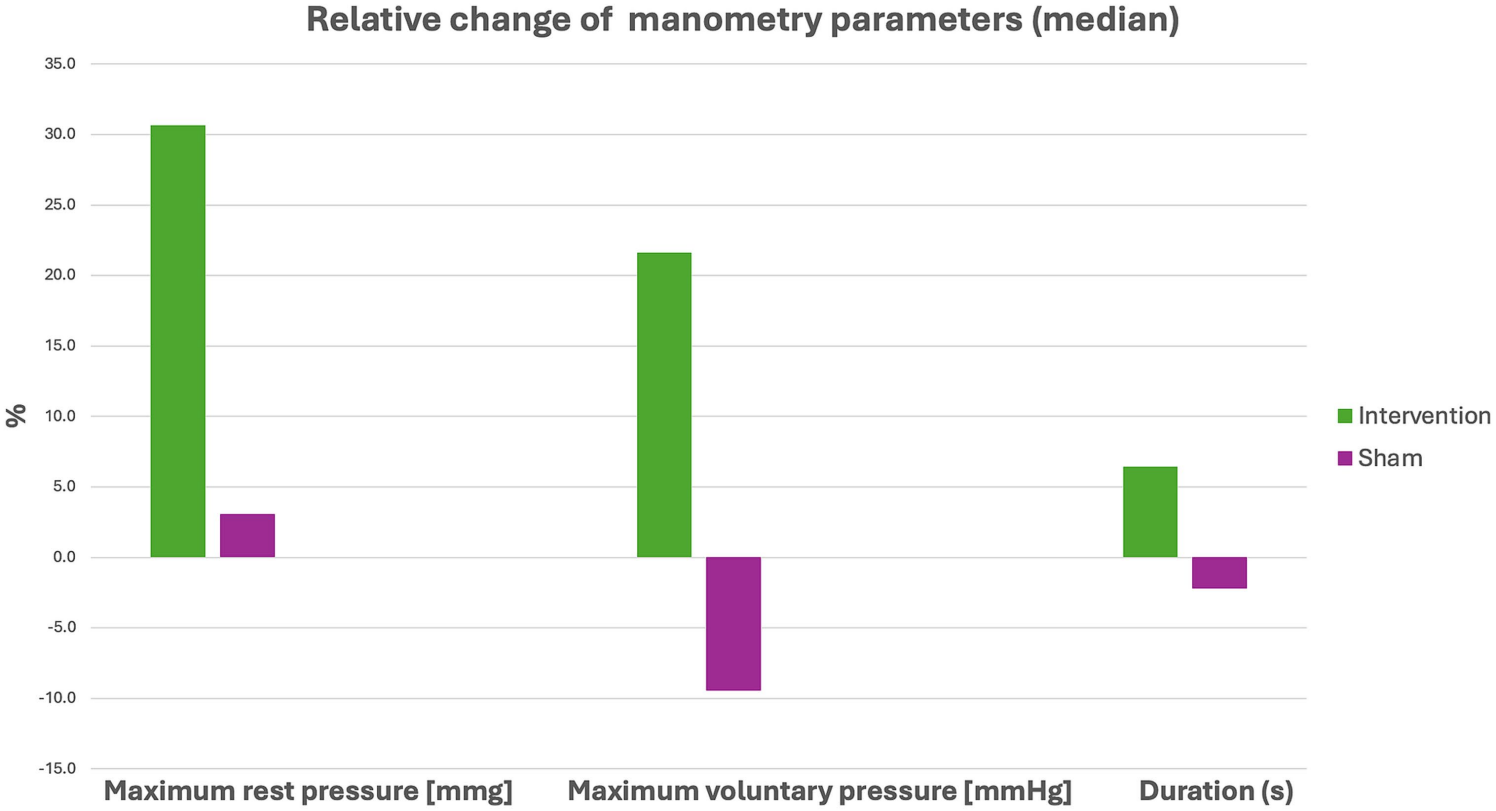
The relative change in objective manometric parameters pre- and post-12-week intervention. Maximum rest pressure and maximum voluntary pressure are presented in mmHg and duration in seconds (s). The figure shows the median values of change for each parameter. No significant differences were found in between group comparison.

The primary endpoints, which were the change in resting maximum pressure and voluntary maximum pressure, were higher in the intervention group than in the sham group, but this difference was not statistically significant. The same was true for the maximum duration of voluntary sphincter squeeze and maximum sphincter pressure during cough. The intervention produced a moderate effect regarding maximum pressure during cough (*r* = 0.41) and a large effect concerning voluntary maximum pressure (*r* = 0.57). The results are summarized in Table 1 and Figure 1.

**Table 1:** Objective manometrical parameters measured by 3D anorectal manometry before and after 12 weeks of home biofeedback training.

[Median, IQR]	Intervention	*p*	*r*	Sham	*p*	*r*
Rest mean pre	46.4 [14.6]	0.68	0.16	36.2 [42.9]	1	<0.1
Rest mean post	49.5 [40.9]			39.2 [29.9]		
Rest maximum pre	51.9 [13.2]	0.56	0.23	42.8 [35.2]	0.68	0.16
Rest maximum post	61.15 [35.7]			50.4 [24.5]		
Voluntary maximum pre	157.75 [108.3]	0.15	0.57	106.8 [44.4]	0.68	−0.16
Voluntary maximum post	173.35 [131.4]			99.4 [83.0]		
Duration pre	6.05 [7.60]	0.68	0.16	8.75 [8.30]	0.46	−0.3
Duration post	8.35 [7.10]			8.30 [9.00]		
Cough pre	108.50 [132.60]	0.31	0.41	98.50 [6.40]	0.56	−0.2
Cough post	124.95 [137.40]			74.90 [37.70]		

Data are presented as median with interquartile range (IQR). Rest mean pressure, rest maximum pressure, maximum voluntary pressure, and cough are presented as mmHg and duration in seconds. Pre: pre intervention; post: post intervention. *p* was calculated with Wilcox signed-rank test, *p* < 0.05 indicated statistical significance. Effect size (*r*) is interpreted as small (>0.1), moderate (>0.3), and large (>0.5).

### Clinical severity outcomes

The secondary endpoints, change in St. Marks score and FISI score, were higher in the intervention group than in the sham group, but with no statistical significance. However St. Marks score was improved with moderate effect in the intervention group (*r* = 0.42). The results are summarized in Table 2.

**Table 2:** Clinical subjective parameters measured by St. Marks score questionary and Fecal Incontince Severity Index (FISI) before and after 12 weeks of home biofeedback training.

Median [IQR]	Intervention	*p*	*r*	Sham	*p*	*r*
St. Marks pre	13.4 [4]	0.34	0.42	9.5 [8]	1	<0.1
St. Marks post	9.5 [6]			10 [13]		
FISI pre	34.5 [7]	0.68	0.18	23.5 [20]	1	<0.1
FISI post	29 [9]			21.5 [13]		

Data are presented as median with interquartile range (IQR). Pre: pre intervention; post: post intervention. *p* was calculated with Wilcox signed-rank test, *p* < 0.05 indicated statistical significance. Effect size (*r*) is interpreted as small (>0.1), moderate (>0.3), and large (>0.5).

### App-based training data

Four patients (two in the intervention group and two in the sham group) were willing to use the provided free application, resulting in six data blocks (four from the intervention group due to the protocol design, in which the sham group received intervention after an initial 12 weeks, plus two sham group data blocks). A total of 62 weekly reports (58% from the intervention group and 42% from the sham group) were uploaded to the app and analyzed. Approximately 15.5 logs per participant (range 2–27) were recorded. The average number of training days was 5.5 (5.6 in the intervention group and 5.4 in the sham group). In the intervention group, 77.4% of trainings were perceived as “not good, not bad,” 21% as good, and 1.6% as bad. In the sham group, all (100%) trainings were perceived as “not good, not bad.” The average logged current used for stimulation was 12.8 mA, with a minimum of 1 mA and a maximum of 38 mA.

## Discussion

To our knowledge, this is the first clinical study with sham control assessing a home-based biofeedback device in a phenotypically well-defined female fecal incontinence group, with objective adherence tracking and exclusion of structural anorectal issues.

Although the efficacy of biofeedback training for fecal incontinence has been extensively evaluated previously (7, 8) evidence on home-based biofeedback therapy for fecal incontinence remains limited. A notable randomized study by Xiang et al. (9) demonstrated that home biofeedback training with local anal electric stimulation can provide comparable clinical outcomes to office-based therapy, although it acknowledged the limitation of interpretation due to the open-label design of the trial. This limitation was inherently addressed by the design of the study presented here, which utilized a sham control. Thus, our study not only offers complementary outcomes to the previously mentioned trial but also provides further data to clarify the mechanism of home biofeedback treatment. As our study shows, home biofeedback with local electrical stimulation leads to improvements in both objective (manometric) and subjective clinical parameters (St. Mark’s score and FISI score), although no statistical significance was found.

As shown in the pilot study by Damin et al. (10), sufficient alleviation of symptoms and improvement of objective manometric parameters can be achieved even after a relatively short training duration, in this case, after 28 training sessions. In comparison, we provided patients with a 12-week program, which should ideally result in 72 training sessions. Based on data from the supporting application, the average number of logged trainings was 66, which we find sufficient, given an adherence rate of 5.5 trainings per week out of the 6 recommended (91.1% adherence). However, not every participant was willing or able to use the mobile application, and since the logged process was self-reporting dependent, we acknowledge that the actual cumulative number of trainings could differ, albeit within an acceptable range. In addition, the stimulation intensity and successful completion of the whole training unit were self-reported, introducing potential recall and reporting bias. Objective device-based adherence logs were unavailable because the study team could not extract the exact session data from the device. Consequently, adherence results should be regarded as indicative of feasibility rather than as definitive objective measures of compliance. Given that biofeedback protocols vary significantly in terms of duration and intensity (11) it is not possible to confidently determine an ideal home biofeedback protocol. On one hand, an insufficient cumulative number of sessions might not be adequate for sphincter function improvement. On the other hand, longer protocol durations—both in terms of individual sessions and total training time—tend to be associated with higher dropout rates. As a study by Vasant et al. reports (12), customizing the intensity of biofeedback therapy and in-home practice could lead to better protocol adherence and, consequently, better treatment outcomes.

Despite the positive results, we recognize the limitations of our study. Firstly, the relatively small number of participants in each study arm. In an exploratory, pilot trial, the sample size was intentionally kept small, based on strong preliminary manometric data indicating a significant increase in anal sphincter pressure after biofeedback. Therefore, the findings should be viewed as hypothesis-generating and helpful for designing a larger, adequately powered randomized control trial. Additionally, our screening-to-allocation ratio was relatively high. This was due to our goal of including only a well-defined, relatively homogeneous cohort to achieve high confidence in the preliminary conclusions from a mechanistic perspective. As was previously reported (13), real-world data suggest the efficacy of home training for fecal incontinence. However, the placebo effect could significantly influence the reported outcomes. Therefore, we conducted a study with sham control on a strictly defined patient cohort.

Furthermore, we acknowledge the absence of structured physiotherapeutic supervision during the home training period. Pelvic floor rehabilitation in its complexity extends beyond simple strengthening of isolated sphincter contractions (14). It also, importantly, incorporates the restoration of coordinated neuromuscular activity involving the pelvic floor, abdominal musculature, diaphragm and postural system (15). Consequently, improvements in anorectal pressure parameters should not be interpreted as definitive evidence of the normalization of pelvic floor coordination. It remains possible that some patients achieved higher squeeze pressure values through compensatory or non-physiological recruitment patterns that were not detectable within the design of the present study. Since, as mentioned above, no physiotherapeutic reassessment of muscular recruitment patterns was conducted during the intervention period, our findings should not be interpreted as evidence that fully unsupervised home rehabilitation can replace structured, professionally led pelvic floor physiotherapy (16). Rather, the current data support the feasibility of home-based electrical stimulation combined with voluntary sphincter training as a potentially valuable adjunct to broader rehabilitation management. Future controlled studies should compare fully home-based, supervised outpatient, and hybrid rehabilitation protocols, incorporating objective assessments of neuromuscular coordination and compensatory muscular recruitment patterns. To provide a guidance for possible future randomized controlled trials (RCTs), we performed a sensitivity analysis in G*Power (v3.1.9.7) (17, 18) to conduct a general power calculation and recommend recruiting 20 paired participants for the RCT.

Lastly, our study lacked follow-up data after a significant period following the end of the treatment protocol. Although at the time of final data evaluation, neither the study nor the clinical team involved had been contacted by patients who experienced a positive effect from the therapy, despite advice to do so in case of recurrence or worsening of symptoms. This suggests that no significant decline in sphincter function was self-noticed within the 3 to 12 months period. We recommend that this observation and evaluation be included in future trials with a home biofeedback device.

## Conclusion

The results of this pilot feasibility study suggest that the use of home-based anal electrical stimulation with voluntary sphincter training may result in favorable trends in anorectal manometric and clinical outcomes among women with fecal incontinence without sphincter structural defects. Although the limited sample size prevented reaching statistical significance, the outcomes demonstrate that this home-supported rehabilitation approach could be utilizable. However, the study was not designed to assess complex neuromuscular coordination or compensatory recruitment patterns. Therefore, it should not be interpreted as evidence that unsupervised home training can replace controlled, therapist-guided pelvic floor therapy. Further randomized controlled studies comparing supervised, home-based, and hybrid rehabilitation modalities should be performed to fully assess the potential of this approach.

## Data Availability

The original contributions presented in the study are included in the article/Supplementary material, further inquiries can be directed to the corresponding author.

## Footnotes

1
randomizer.org
