# Supplementary material for: Home-based biofeedback with local anal electrical stimulation for fecal incontinence in women without sphincter structural defects
Source: Front Med (Lausanne). 2026 Jun 12;13:1835226. doi: 10.3389/fmed.2026.1835226 (PMC13303362; doi:10.3389/fmed.2026.1835226)
Supplement: Supplementary file 1 [file Supplementary_File_1.doc]

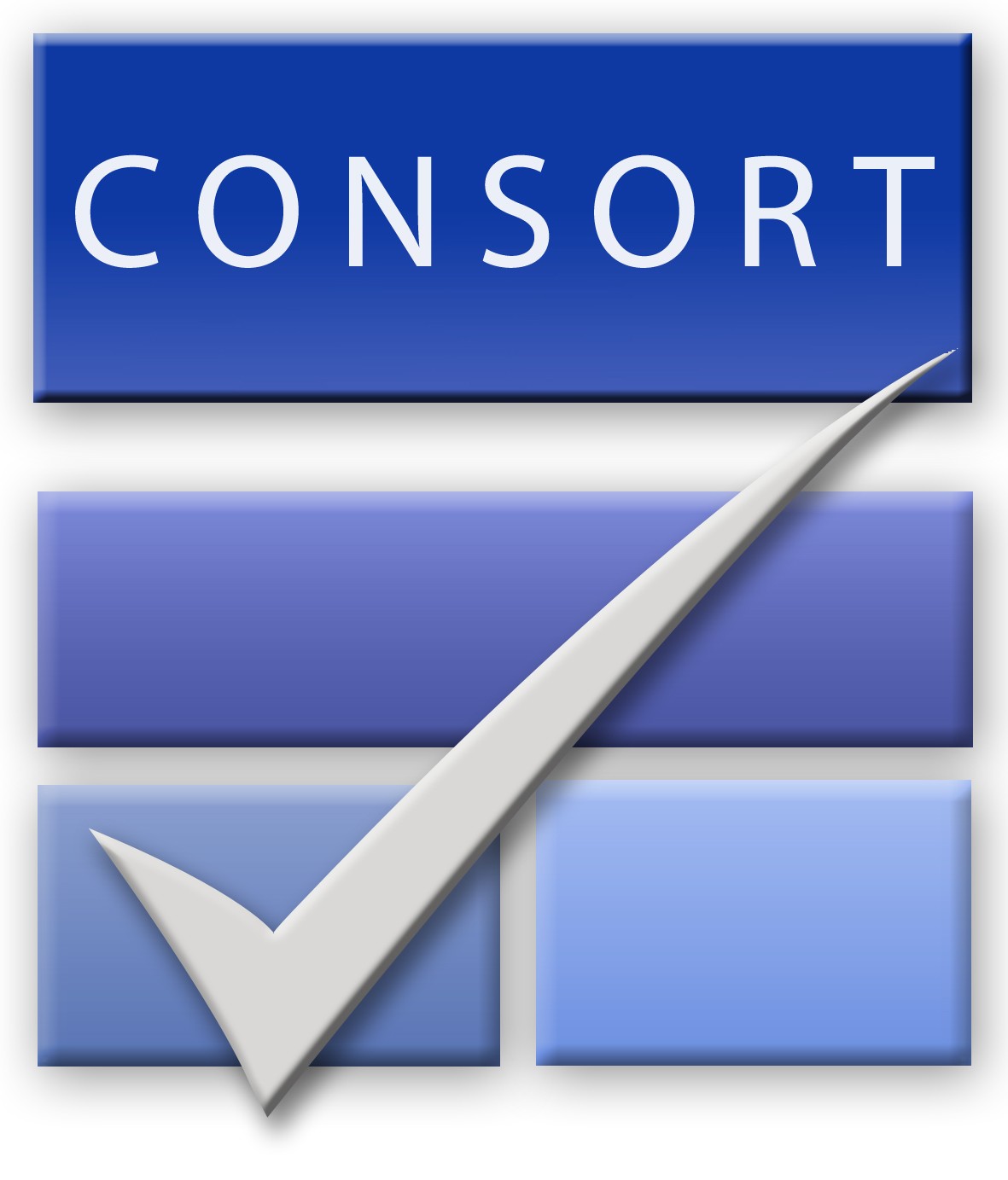
**CONSORT 2010 checklist of information to include when reporting a pilot or feasibility randomized trial in a journal or conference abstract**

| **Item** | **Description** | **Reported on line number** |
| --- | --- | --- |
| Title | Identification of study as randomised pilot or feasibility trial | **1** |
| Authors * | Contact details for the corresponding author | **14** |
| Trial design | Description of pilot trial design (eg, parallel, cluster) | **73** |
| Methods |  |  |
| Participants | Eligibility criteria for participants and the settings where the pilot trial was conducted | **137** |
| Interventions | Interventions intended for each group | **196,199** |
| Objective | Specific objectives of the pilot trial |  |
| Outcome | Prespecified assessment or measurement to address the pilot trial objectives** | **129-131** |
| Randomization | How participants were allocated to interventions | **156** |
| Blinding (masking) | Whether or not participants, care givers, and those assessing the outcomes were blinded to group assignment | **137** |
| Results |  |  |
| Numbers randomized | Number of participants screened and randomised to each group for the pilot trial objectives** | **234** |
| Recruitment | Trial status† |  |
| Numbers analysed | Number of participants analysed in each group for the pilot objectives** | **240** |
| Outcome | Results for the pilot objectives, including any expressions of uncertainty** | **250** |
| Harms | Important adverse events or side effects | **245** |
| Conclusions | General interpretation of the results of pilot trial and their implications for the future definitive trial | **506** |
| Trial registration | Registration number for pilot trial and name of trial register | **142** |
| Funding | Source of funding for pilot trial | **552** |

Citation: Eldridge SM, Chan CL, Campbell MJ, Bond CM, Hopewell S, Thabane L, et al.

**this item is specific to conference abstracts*

***Space permitting, list all pilot trial objectives and give the results for each. Otherwise, report those that are a priori agreed as the most important to the decision to proceed with the future*

*definitive RCT.*

*†For conference abstracts.*
